# UHRF1 Induces Metastasis in Thyroid Cancer

**DOI:** 10.1155/2022/7716427

**Published:** 2022-08-13

**Authors:** Bo-Hua Kuang, Guo-He Lin, Quentin Liu, Bi-Cheng Wang

**Affiliations:** ^1^Cancer Center, Union Hospital, Tongji Medical College, Huazhong University of Science and Technology, Wuhan 430022, China; ^2^Department of Oncology, The Second Affiliated Hospital of Anhui Medical University, Hefei, Anhui 230601, China; ^3^State Key Laboratory of Oncology in South China, Collaborative Innovation Center for Cancer Medicine, Cancer Center, Sun Yat-Sen University, Guangzhou 510060, China; ^4^Institute of Cancer Stem Cell, Dalian Medical University, Dalian 116044, China

## Abstract

**Background:**

Ubiquitin-like with PHD and ring-finger domain 1 (UHRF1) has been defined as an oncogene in tumor cells. However, the role of UHRF1 in mediating metastasis in thyroid cancer remains unexplored. In this study, we aimed to investigate the metastatic function and the potential mechanisms of UHRF1 in thyroid cancer.

**Methods:**

Transwell assays were used to detect the metastatic capability of thyroid cancer. Dual-luciferase reporter assays were applied to examine the activation of transcription factors. Coimmunoprecipitation assays and immunofluorescence staining assays were used to elucidate the potential mechanisms of UHRF1 in promoting the metastasis of thyroid cancer.

**Results:**

In this study, we found that overexpression of UHRF1 promoted the metastasis of papillary thyroid cancer cells, and suppression of UHRF1 decreased the metastasis of anaplastic thyroid cancer cells. Regarding the signaling pathway in regulating metastasis, UHRF1 directly combined and activated the transcription factor c-Jun/AP-1 in the nucleus, subsequently increasing the transcription of IL-6 and MIF.

**Conclusion:**

Our results suggest that UHRF1 could induce the metastasis of thyroid cancer, and the potential signaling pathway might be that UHRF1 activates c-Jun/AP-1 to increase the expression of IL-6 and MIF. These findings provide a novel mechanism of UHRF1 and illustrate that UHRF1/AP-1 complex could be a potential therapeutic target for patients with thyroid cancer.

## 1. Introduction

Thyroid tumors mainly include four subtypes, papillary, follicular, medullary, and anaplastic thyroid cancers. Most thyroid cancer patients have an extremely well prognosis, while regional or distant metastases are the primary causes of the shorter survival time and mortality of thyroid cancer. However, the mechanisms of thyroid cancer cell metastasis remain unclear [1, 2]. In terms of papillary thyroid cancer (PTC), patients could have been cured after surgery, but cancer cells could spread throughout the thyroid gland and to the regional lymph nodes and distant organs [3–6]. Once patients with anaplastic thyroid cancer (ATC) are diagnosed, they are at stage IV of disease, and tumor cells have already spread to distant organs. Our previously published study showed that UHRF1 was significantly overexpressed in PTC and ATC compared with normal thyroid cancer tissues, and suppressing UHRF1 decreased the proliferation of ATC and induced differentiation [7].

Several studies have revealed that UHRF1 was an oncogene and could promote the development of cancer cells. Moreover, UHRF1 could be considered a potential biomarker and a therapeutic target in cancer treatments [8–10]. For example, large-scale cancer genomic data analyses revealed that lung adenocarcinoma or acute myeloid leukemia patients with a high expression level of UHRF1 had significantly worse survival outcomes [11, 12]. In addition, UHRF1 accelerated the metastasis of colorectal cancer [13]. The expression level of UHRF1 protein in primary hepatocellular carcinoma had a prognostic significance in predicting the development of distant metastasis [9]. Increasing evidence suggested that UHRF1 promoted invasion and proliferation via mediating hypermethylation of tumor suppressor genes (e.g., p16^INK4A^, PPARG, BRCA1, and PML) [8, 14]. However, the role and molecular mechanisms of UHRF1 in driving thyroid cancer metastasis remain unknown.

Therefore, understanding how UHRF1 promotes thyroid tumor cell metastasis might be essential to retard the progression of cancer cells. Here, we conducted this study to investigate the metastatic function and mechanisms of UHRF1 in thyroid cancer.

## 2. Materials and Methods

### 2.1. Cell Lines and Cell Culture Conditions

The papillary thyroid cancer cell line BCPAP and the anaplastic thyroid cancer cell line 8505c were purchased from Guangzhou Jenniobio Biotechnology Co., Ltd. BCPAP and 8505c cells were, respectively, maintained in DMEM (Invitrogen) and RPMI 1640 (Gibco) supplemented with 1% antibiotics and 10% fetal bovine serum (Gibco) at 37°C in a humidified chamber containing 5% CO_2_. All cell lines were authenticated by short tandem repeat (STR) DNA fingerprinting, and mycoplasma testing was operated by the Medicine Lab of Forensic Medicine Department of Sun Yat-sen University (Guangzhou, China).

### 2.2. UHRF1 and Mutant Plasmid Construction

Human UHRF1 was cloned into the pcDNA6-myc-His B (pcDNA6B) vector. UHRF1 mutants were generated using a One Step Cloning kit CloneExpress® II (Vazyme) according to the manufacturer's instructions.

### 2.3. Quantitative Real-Time Polymerase Chain Reaction (qPCR)

Total RNA was extracted by using the RaPure Total RNA kit (Magen), which was used to generate cDNA by using TranScript® All-in-One First-Strand cDNA Synthesis SuperMix for qPCR (One-Step gDNA Removal) (TransGen Biotech). qPCR was performed using the Bio-Rad sequence system CFX96 with ChamQ™ SYBR® qPCR Master Mix (2×) (Vazyme) as recommended by the manufacturer. The primers used were as follows: IL-6F: 5′-TCCAGTTGCCTTCTCCC-3′, R: 5′-GCCTCTTTGCTGCTTTC-3′; MIF F: 5′-CGCAGAACCGCTCCTACA-3′, R: 5′-GAGTTGTTCCAGCCCACATT-3′.*β*-actin (F: 5′- CATCCGCAAAGACCTGTACG-3′, R: 5′- CCTGCTTGCTGATCCACATC-3′) was used as the internal control. qPCR results were analyzed and converted to fold changes.

### 2.4. Western Blot (WB) Analysis

Cells were lysed by using RIPA buffer on ice for 30 min. The Bradford dye method was used to detect the protein concentration. Equal amounts of cell protein were subjected to electrophoresis in 10% SDS-PAGE gels and then transferred to nitrocellulose filter (NC) membranes (Millipore) for antibody blotting. The membranes were blocked in 5% BSA (TBS-T buffer) at room temperature for 1 h and then incubated with a primary antibody (5% BSA) at 4°C for 24 h. The following antibodies were used as the primary antibodies: UHRF1 (Abcam), 6 × His-tag (Proteintech), Flag-tag (Proteintech), GAPDH (Proteintech), and *β*-actin (Cell Signaling Technology). The secondary antibody was Horseradish peroxidase-conjugated goat antimouse IgG (Pierce).

### 2.5. Coimmunoprecipitation (Co-IP) Assay

BCPAP cells were lysed with IP lysis buffer (20 mM Tris-HCl pH 8.0, 150 mM NaCl, 0.5% NP-40, 10% Glycerol). The extract was collected and rotated at 4°C for 20 min and then spun at 14,000 g for 20 min at 4°C; 10% was kept for input, while the rest was incubated with the His-tag or Flag-tag antibody for 3 h at 4°C. Protein *A*/*G* beads (Roche) were washed 3 times with PBS and added for 16 h incubation at 4°C. The beads were washed with IP lysis buffer 3 times, and the bound proteins were detected with the His-tag or Flag-tag antibodies.

### 2.6. Cell Invasion Assays

For the transwell invasion assay, the upper chambers of the Transwell plates (Millipore) were precoated with Matrigel (BD Biosciences) (1 : 8 mixed with PBS) at 37°C for 30 minutes. Next, 1 × 10^5^ BCPAP cells or 2 × 10^4^ 8505c cells were resuspended in 200 *μ*l serum-free DMEM/1640 and placed in the upper chambers, and 580 *μ*l of culture medium (DMEM/1640 with 10% FBS) was added to the lower chambers. The cells were then incubated at 37°C with 5% CO_2_ for 30 hours. Migrated cells were stained with crystal violet and counted. Results were shown as the average from at least three independent experiments.

### 2.7. Dual-Luciferase Reporter Assay

BCPAP cells were seeded on 12-well plates and transfected with the 500 ng luciferase reporter construct (the AP-1-luciferase reporter and the NF-*κ*B-luciferase reporter were kindly provided by Professor Shu) [15, 16], 100 ng Renilla, 500 ng URHF1-His, and 500 ng c-Jun Flag. Cells were collected 48 h later after transfection. The dual-luciferase reporter assay (Promega) was used to perform the reporter assay according to the manufacturer's instructions.

### 2.8. Immunofluorescence Staining

Cells plated on slides were transfected with UHRF1-His and c-Jun-Flag for 48 h. Then, the cells were fixed in 4% paraformaldehyde at room temperature (RT) for 12 min and permeabilized in 0.5% TritionX-100 (PBS) for 5 min. The slides were blocked with 3% BSA (PBS) for 1 h at RT. After that, the cells were incubated with primary antibodies, Flag-tag (Proteintech) and His-tag (Proteintech), at RT for 2 h, followed by Alexa Fluor 488-conjugated and 546-conjugated secondary antibodies (Invitrogen). The cells counterstained with DAPI (1 *μ*g/mL; Sigma) were visualized using a confocal microscope (Olympus).

### 2.9. Public Database Analysis and Statistical Analysis

The UCSC dataset (https://www.genome.ucsc.edu) was used to analyze the binding motif of promoters. Experiments were performed in triplicate at least three times. Unless otherwise indicated, results were shown as the mean ± SD of three independent experiments. Statistics were calculated by GraphPad Prism 6 software or SPSS software (version 16.0) by two-tailed Student's *t-*test. A *p* value < 0.05 (^*∗*^*p* < 0.05, ^*∗∗*^*p* < 0.01, ^*∗∗∗*^*p* < 0.001) was considered statistically significant.

## 3. Results

### 3.1. UHRF1 Increased the Metastasis of Thyroid Cancer

**Figure 1:**
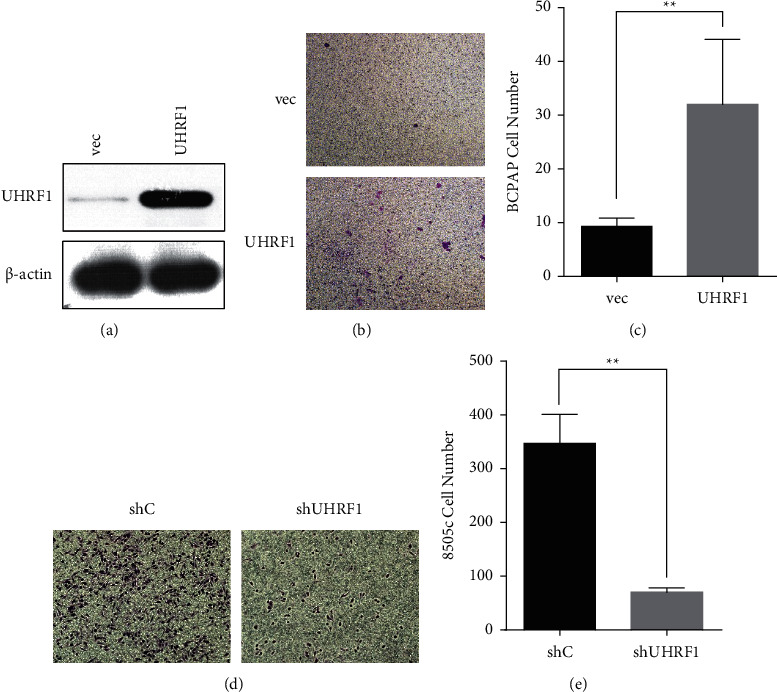
Overexpressed UHRF1 increased the metastasis of thyroid cancer. (a) BCPAP cells were transfected with UHRF1 or a vector control. Validation of the UHRF1 overexpression by the western blot is shown. (b, c) The transwell invasion assay of BCPAP transfected with UHRF1 or an empty vector was performed. (d, e) The transwell invasion assay of the anaplastic thyroid cancer cell line 8505c transfected with shC or shUHRF1 was performed. A representative experiment is shown in triplicate along with the mean ± SD in D-G, ^*∗∗*^*p* < 0.01, ^*∗∗∗*^*p* < 0.001 by two-tailed Student's *t*-test.

In order to identify whether UHRF1 induces metastasis in thyroid cancer, we overexpressed UHRF1 in the well-differentiated thyroid cancer cell line BCPAP. The UHRF1 expression level was detected by WB (Figure 1(a)). UHRF1 upregulation resulted in a significant increase in invasion in thyroid cancer cells (Figures 1(b)–1(c)). Conversely, suppression of UHRF1 in the anaplastic thyroid cancer cell line 8505c significantly inhibited the invasion capability (Figures 1(d)–1(e)). The efficacy of shRNA plasmids had been certificated in our previously published study [7].

### 3.2. UHRF1 Increased the Transcriptional Activity of c-Jun/AP-1

Cancer-related inflammation has been certificated to promote regional and distant metastasis [17, 18]. In the tumor microenvironment, the transcription factors AP-1 [19–21] and NF-*κ*B [22] played a crucial role in inflammation-related metastasis. In our previous published study [7], we found that UHRF1 suppression significantly decreased the expression of cytokines, including IL-6, IL-8, and TNF-*α*/*β*. Notably, IL-6, IL-8, and TNF-*α*/*β* could be regulated by both AP-1 and NF-*κ*B [15, 23, 24]. Moreover, in the analysis of the UCSC dataset (https://www.genome.ucsc.edu), we found that AP-1 could directly bind to the promoters of IL-6 and IL-8 (Supplementary Figure S1). Thus, we mainly detected the effects of UHRF1 on c-Jun/AP-1 and NF-*κ*B transactivation functions.

**Figure 2:**
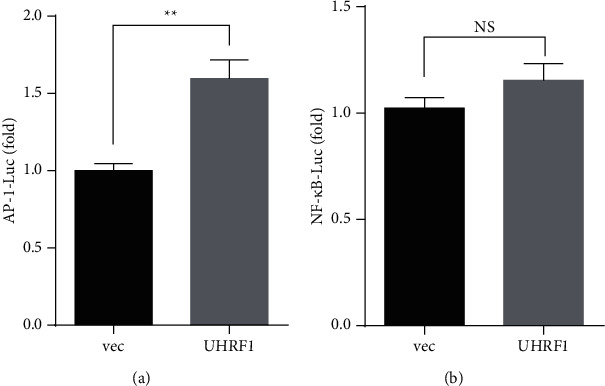
UHRF1 activated the transcriptional function of c-Jun/AP-1. UHRF1 or a vector control was transiently cotransfected with the indicated luciferase reporter plasmid and pRL-TK encoding Renilla luciferase as an internal normalized control into BCPAP cells, and then, c-Jun/AP-1 (a) and NF-*κ*B (b) luciferase activities were detected. Data are the mean ± SD of three independent experiments, NS, not significant, ^*∗∗*^*p* < 0.01, ^*∗∗∗*^*p* < 0.001 by two-tailed Student's *t*-test.

BCPAP cells were cotransfected with c-Jun/AP-1-Luc and UHRF1 constructs. Results showed that the c-Jun/AP-1 luciferase activity was significantly increased by UHRF1 (Figure 2(a)). However, UHRF1 failed to activate the NF-*κ*B transactivation function (Figure 2(b)).

**Figure 3:**
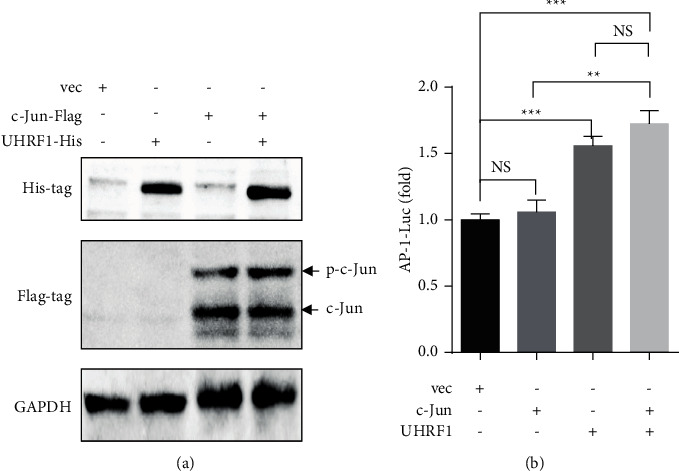
UHRF1 increased the activation of c-Jun/AP-1. (a) BCPAP cells were cotransfected with UHRF1 and c-Jun/AP-1. The western blot was used to detect the expression of c-Jun and p-c-Jun. (b) BCPAP cells were cotransfected with c-Jun/AP-1 luciferase reporter, UHRF1, and c-Jun. Luciferase assay results are shown as a fold change of luciferase normalized to the c-Jun/AP-1 reporter and the vector plasmid alone. Data are the mean ± SD of three independent experiments, NS, not significant, ^*∗*^*p* < 0.05, ^*∗∗*^*p* < 0.01, ^*∗∗∗*^*p* < 0.001 by two-tailed Student's *t*-test.

Subsequently, UHRF1 and c-Jun/AP-1 were cotransferred into the BCPAP cells. We found that the phosphorylation levels of c-Jun/AP-1 (p-c-Jun) were elevated by UHRF1, but c-Jun/AP-1 protein levels showed no changes (Figure 3(a)). We further conducted AP-1-Luc assays to confirm the increase of p-c-Jun by UHRF1. Compared with the c-Jun/AP-1 group, overexpression of both c-Jun/AP-1 and UHRF1 induced a modest 1.5-fold to 2-fold activation of the c-Jun/AP-1 luciferase reporter (Figure 3(b)).

Based on these results, we suggested that UHRF1 could promote the c-Jun/AP-1 transactivation.

### 3.3. UHRF1 Interacted with c-Jun/AP-1

**Figure 4:**
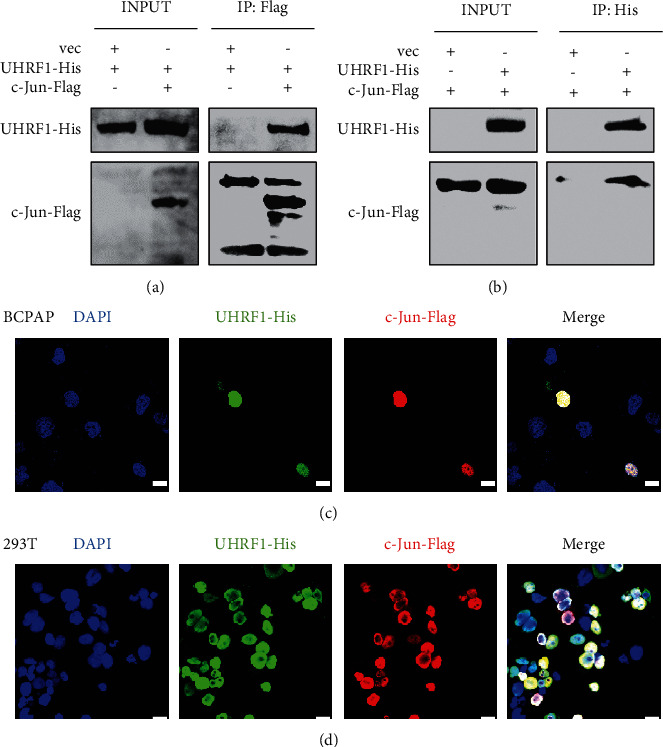
UHRF1 combined directly with c-Jun/AP-1 in the nucleus. BCPAP cells were transfected according to the panel labels. The co-IP assay was performed using either an anti-Flag antibody to pull down Flag-tagged c-Jun proteins (a) or an anti-His antibody against UHRF1-His protein (b). BCPAP and 293T cells were cotransfected with UHRF1-His and c-Jun-Flag. Immunofluorescence staining was performed to visualize the colocalization of UHRF1 (green) and c-Jun (red). Nuclear DNA was stained with DAPI (blue) (c, d). The scale bar represents 100 *μ*m.

Co-IP and reciprocal co-IP assays were performed for further confirmation. BCPAP cells were cotransfected with His-tagged UHRF1 and Flag-tagged c-Jun/AP-1. In Figures 4(a) and 4(b), we found that c-Jun/AP-1 was directly combined with UHRF1.

IF assays were applied further to verify the interaction between UHRF1 protein and c-Jun protein. His-tagged UHRF1 and Flag-tagged c-Jun were cotransfected with BCPAP cells. 293T cells were applied owing to the high transduce efficiency. As shown in Figures 4(c) and 4(d), UHRF1 was localized with c-Jun in the nucleus.

### 3.4. UHRF1-c-Jun/AP-1 Complex Increased the Transcription of Inflammation/Metastasis-Related Cytokines

**Figure 5:**
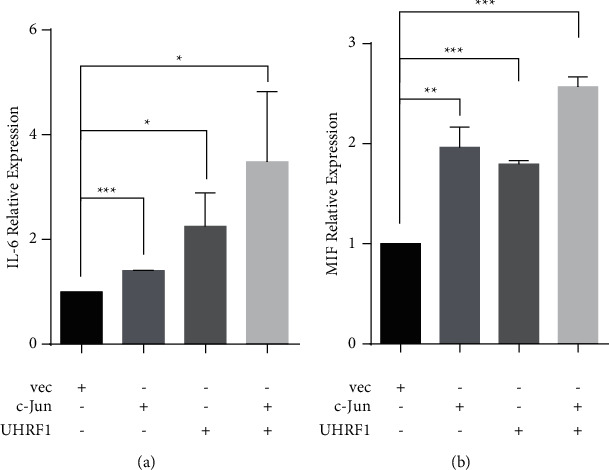
UHRF1-c-Jun/AP-1 complex enhanced the expression of IL-6 and MIF. (a) Validation of IL-6 expression by qPCR when BCPAP cells were transfected with UHRF1-His and c-Jun-Flag (^*∗*^*p* < 0.05, ^*∗∗*^*p* < 0.01, ^*∗∗∗*^*p* < 0.001 by two-tailed Student's t-test). (b) Validation of MIF expression by qPCR in BCPAP cells as indicated (NS, not significant, ^*∗∗*^*p* < 0.01, ^*∗∗∗*^*p* < 0.001 by two-tailed Student's *t*-test).

Cytokines secreted by thyroid cancer cells and infiltrating immune cells in the tumor microenvironment promoted proliferation, migration, angiogenesis, and metastasis [25]. To study how UHRF1 promoted thyroid cancer cell metastasis, we analyzed the transcription levels of key regulators of immunoregulatory cytokine interleukin 6 (IL-6) and the macrophage migration inhibitory factor (MIF) in BCPAP by qPCR. The results showed that the mRNA levels of IL-6 and MIF were significantly higher in cells cotransfected with UHRF1 and c-Jun/AP-1 than those of UHRF1 or c-Jun alone (Figure 5). These data suggested that the transcriptions of cytokines IL-6 and MIF were increased by UHRF1-c-Jun/AP-1 complex.

## 4. Discussion

In this study, we demonstrated that UHRF1 promoted the metastasis of thyroid cancer cells through a potential mechanism that UHRF1 directly bound and activated c-Jun/AP-1. Accordingly, UHRF1-c-Jun/AP-1 complex could be a potential treatment target for thyroid cancer.

UHRF1 has become an important prognostic biomarker and a vital cancer therapeutic target. Our previous study confirmed that UHRF1 protein was overexpressed in thyroid cancer cells [7], which meant UHRF1 might also be a critical gene for thyroid cancer in predicting survival and treatment responses. Although results have indicated that suppression of UHRF1 could decrease the metastasis of thyroid cancer cells, it is more important to understand the underlying mechanisms that UHRF1 promotes the metastasis. This study identified UHRF1-induced activation of c-Jun/AP-1 and UHRF1-promoted transcription of inflammation/metastasis-related cytokines, which favored cancer cell migration and invasion. Moreover, knocking down UHRF1 suppressed the invasion. It is yet to be seen how UHRF1-mediated overexpression of cytokines causes inflammatory reaction and metastasis in thyroid cancer. Further experiments are needed to detect the accurate and solid pathway between UHRF1-c-Jun/AP-1 and metastasis.

The poorly differentiated cancer, ATC, overexpresses UHRF1 and has a highly distant metastatic capability, while PTC, a type of well-differentiated cancer, develops slowly and has a good prognosis. Our previously published study confirmed that UHRF1 promoted the dedifferentiation of thyroid cancer. During the process, well-differentiated thyroid cancer cells are transferred into poorly differentiated cells. Further, metastasis-related cytokines were positively regulated by UHRF1. According to our previous and present results in thyroid cancer, UHRF1 should be an essential gene in dedifferentiation and metastasis [7, 26–28].

Throughout cancer development, the main published downstream targets of UHRF1 were tumor suppressor genes. The regulation depended on the cooperation of DNA methyltransferase 1 (DNMT1) and UHRF1 via the SRA domain to the hemimethylated sites [29–31]. For instance, Kelch-like ECH-associated protein 1 (KEAP1) was absent in pancreatic ductal adenocarcinoma (PDAC) and negatively correlated with the malignancy of PDAC. Depleting UHRF1 reduced KEAP1 promoter methylation, leading to the restoration of KEAP1 protein [32].

However, driver oncogenes have not been found to be regulated by UHRF1. c-Jun/AP-1 was targeted for proteasomal degradation by diverse E3-ligase complexes (Fbw7, Itch, and COP1) [33–37]. Given that UHRF1 contained a RING domain, it was conceivable that UHRF1 might interact with c-Jun/AP-1. In this study, we found that the oncogene c-Jun/AP-1 directly combined with UHRF1 and could be transcriptionally activated by UHRF1. For the first time, we reported that the driver oncogene c-Jun/AP-1 could be regulated by UHRF1. This discovery might indicate a new mechanism for UHRF1 in tumor development.

The tumor microenvironment is strongly correlated with tumor metastasis [38]. Infiltrated lymphocytes induce cancer cells to migrate into the vessels and then transfer to distant organs [39]. Cytokines secreted by cancer cells mediate the aggregation of lymphocytes in the tumor microenvironment [40]. Based on these studies, the signaling pathway that UHRF1 plus c-Jun/AP-1 increased the expression of IL-6 and MIF might be a potential key mechanism of thyroid cancer metastasis.

Finally, we deduced that UHRF1-c-Jun/AP-1 complex could promote metastasis in thyroid cancer. Targeting this pathway might provide more insights into treatment strategies for thyroid cancer patients.

## Data Availability

The raw data are available from the corresponding author upon reasonable request.

## Supplementary Materials

Supplementary Materials Figure S1: UCSC dataset analyses of c-Jun/AP-1 binding to the promoters of IL-6 and.
