# Supplementary figures and images for: UHRF1 Induces Metastasis in Thyroid Cancer
Source: J Oncol. 2022 Aug 13;2022:7716427. doi: 10.1155/2022/7716427 (PMC9392644; doi:10.1155/2022/7716427)

**Figure S1** UCSC dataset analyses of c-Jun/AP-1 binding to the promoters of IL-6 and IL-8.


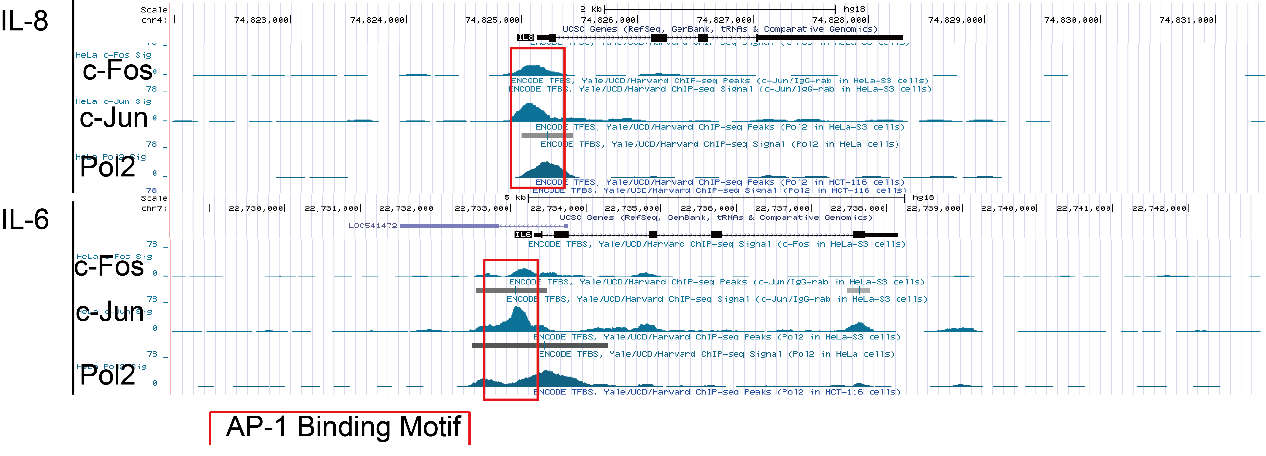

Supplement: Supplementary Materials — Figure S1: UCSC dataset analyses of c-Jun/AP-1 binding to the promoters of IL-6 and. [file 7716427.f1.docx]
